# African Swine Fever Virus Strain Georgia 2007/1 in *Ornithodoros erraticus* Ticks

**DOI:** 10.3201/eid1806.111728

**Published:** 2012-06

**Authors:** Adriana V. Diaz, Christopher L. Netherton, Linda K. Dixon, Anthony J. Wilson

**Affiliations:** The Royal Veterinary College, London, United Kingdom (A.V. Diaz);; Institute for Animal Health, Pirbright, Surrey, United Kingdom (A.V. Diaz, C.L. Netherton, L.K. Dixon, A.J. Wilson)

**Keywords:** Asfarviridae, African swine fever virus, African swine fever, infectious disease vectors, arthropod vectors, arachnid vectors, ticks, Argasidae, Ornithodoros, infectious diseases, emerging, swine, pigs, vector-borne infections

**To the Editor:** African swine fever virus (ASFV) causes a notifiable disease in domestic pigs for which no treatment or vaccine is available, resulting in a mortality rate of <100%. In 2007 ASFV was detected in the Caucasus region, first in Georgia and subsequently in Armenia, Azerbaijan, and many parts of Russia, including regions that border other countries in Europe and Asia (*1*).

Most field strains of ASFV can persistently infect *Ornithodoros* ticks, including the species *O. erraticus* in southern Europe (*2*), and ASFV has been isolated from ticks collected >5 years after the last confirmed case in an outbreak (*3*). These ticks can feed on alternative hosts, evade eradication attempts (such as acaricide application and flamethrowers), and survive for up to 15 years (*1*). Although *Ornithodoros* species have been reported in the Caucasus region, their distribution is not well known (*1*). It is also not known if the Georgia 2007/1 ASFV strain responsible for continuing outbreaks in the Caucasus region can replicate in ticks. Thus, we conducted a study to determine whether the Georgia 2007/1 isolate of ASFV can replicate in *Ornithodoros* ticks.

*O. erraticus* ticks from Alentejo, Portugal (provided by Fernando Boinas, Universidade Técnica de Lisboa in Lisbon, Portugal) were sorted into groups of 10 adults or fifth-instar nymphs, placed into 60-mL containers covered with nylon cloth (16-cm mesh), and maintained at 85% relative humidity and 27°C for 18 months without feeding. Heparinized pig blood containing antibacterial drugs and fungicide (10 µL of streptomycin [10,000 IU/mL], 10 µL of amphotericin B [250 µg/mL], and 5 µL of neomycin [10 mg/mL 0.9% NaCl]/mL of blood) was mixed with the Georgia 2007/1 isolate (*4*) or the OUR T88/1 isolate (*5*) as a positive control to obtain virus titers of 4 log_10_ or 6 log_10_ 50% hemadsorbing doses (HAD_50_)/mL blood. These titers were within the observed range in naturally infected pigs (*6*), and thus simulated the field situation.

Ticks were fed infected blood by using a Hemotek membrane-feeding system (Discovery Workshops, Accrington, UK). Meal reservoirs were covered with stretched Parafilm that was wiped with a thin film of uninfected blood to encourage feeding. Pots of ticks were placed on the membrane and allowed to feed for 20 minutes.

Immediately after and 3, 6, 9, and 12 weeks after feeding, 10 ticks from each feeding group were killed by freezing in dry ice. After being washed with a detergent solution and phosphate-buffered saline, ticks were placed individually in tubes with 200 µL of RPMI medium (Sigma-Aldrich Company Ltd., Gillingham, UK), a 3 mm-diameter stainless steel ball (Dejay Distribution Ltd., Launceston, UK), and 1-mm silicon carbide particles (Stratech Scientific Ltd, Newmarket, UK). They were then homogenized by shaking for 5 cycles of 3 minutes at 25-Hz frequency using a TissueLyser (QIAGEN, Valencia, CA, USA). To complete a 1-mL volume, 800 µL of RPMI medium was added to the tubes after centrifuging 2× for 30 seconds at 2,000 rpm. Supernatants were transferred to fresh tubes and centrifuged for 5 minutes at 1,000 × *g*.

Virus titers were estimated on porcine bone marrow cells (*7*) and expressed as log_10_ HAD_50_ per tick. Previous studies suggest that it takes 3–4 weeks for ticks to completely digest and clear ingested blood and that virus isolated after this period is due to viral replication (*5**,**6*). A general linear model, fitted via maximum likelihood, was used to assess the effects of isolate, dose, time after feeding, and interaction between isolate and time after feeding on the viral titer in the tick. Confidence intervals were calculated by profile likelihood.

Results showed that the Georgia 2007/1 strain can replicate in the *O. erraticus* tick. We recovered virus titers of <1.8 to >9.8 log_10_ HAD_50_ per tick. Ticks that fed on blood containing 6 log_10_ HAD_50_ ASFV on average had virus titers 2.15 log_10_ HAD_50_ higher than those for ticks that fed on blood containing 4 log_10_ HAD_50_/mL. Over time, the average titer for both isolates increased at an estimated rate of 0.65 log_10_ HAD_50_/week, indicating replication. Statistical analysis suggested that immediately after feeding, ticks fed on the Georgia 2007/1 strain contained 1.36 log_10_ HAD_50_ less virus than those fed on the OUR T88/1 isolate, but we detected no statistically significant difference in the replication rates of the 2 isolates. Parameter estimates are shown in the Table and the model fit is shown in the Figure.

**Table:** General linear model of the effects of different parameters on the titer of ASFV in experimentally infected *Ornithodoros erraticus* ticks*

Parameter	Maximum likelihood estimator (95% CI)
Constant	1.4985 (0.7084 to 2.2610)†
ASFV strain	–1.3620 (–2.4007 to –0.3482)†
Dose	2.1538 (1.5889 to 2.7316)†
Time after feeding (effect per week)	0.6494 (0.5546 to 0.7481)†
Isolate–time interaction	–0.0025 (–0.1400 to 0.1363)‡

*Ticks were fed pig blood with 4 log_10_ or 6 log_10_ HAD_50_/mL ASFV strain Georgia 2007/1 or strain OURT88/1. ASFV, African swine fever virus; HAD_50_, 50% hemadsorbing doses. †Statistically significant, p<0.01. ‡Not significant (p>0.05).

**Figure:**
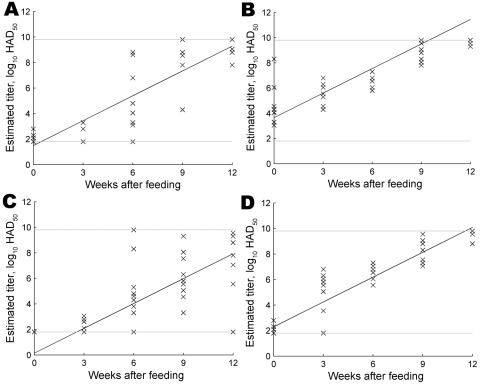
Predicted regression for each isolate–dose combination is shown. A) Ticks fed on African swine fever virus (ASFV) strain OUR T88/1 at 4 log_10_ 50% hemadsorbing doses (HAD_50_)/mL. B) Ticks fed on ASFV strain OUR T88/1 at 6 log_10_ HAD_50_/mL. C) Ticks fed on ASFV strain Georgia 2007/1 at 4 log_10_ HAD_50_/mL. D) Ticks fed on ASFV strain Georgia 2007/1 at 6 log_10_ HAD_50_/mL. Crosses indicate experimental results, and solid line indicates model prediction. Dashed horizontal lines show the limits of the tissue culture sensitivity (lower limit 1.8 log_10_ HAD_50_ and upper limit 9.8 log_10_ HAD_50_).

The whole-tick titers reported in this study are consistent with those from previous studies (*5**,**6*). However, >9 weeks after the ticks fed, we observed higher titers than those reported (*5*), and many results showed >9.8 log_10_ HAD_50_ per tick. The TissueLyser may have been more effective at releasing ASFV from tick tissues than previously used methods. Our results suggest that virus replication within the ticks began by 3 weeks after feeding on infected blood; this timing is consistent with that in previous studies (*5**,**8**,**9*).

We observed high viral titers for >12 weeks after infection. Previous studies showed that ASFV can persist at high titers for 20 weeks after infection (*10*). We demonstrated that ASFV Georgia 2007/1 isolate can replicate efficiently in ticks. This finding highlights the importance of clarifying the distribution of *Ornithodoros* species ticks in the Russian Federation and Caucasus region and the relationship of these ticks to species susceptible to ASFV.

## References

[1] European Food Safety Authority. EFSA Panel on Animal Health and Welfare; scientific opinion on African swine fever. EFSA Journal. 2010;8:1556. 10.2903/j.efsa.20101556

[2] Sanchez-Botija C. Reservoirs of ASFV: a study of the ASFV in arthropods by means of the haemadsorption test. Bull Off Int Epizoot. 1963;60:895–9.

[3] Boinas FS, Wilson AJ, Hutchings GH, Martins C, Dixon LJ. The persistence of African swine fever virus in field-infected *Ornithodoros erraticus* during the ASF endemic period in Portugal. PLoS ONE. 2011;6:e20383. 10.1371/journal.pone.0020383 21655242 PMC3105027

[4] Rowlands RJ, Michaud V, Heath L, Hutchings G, Oura C, Vosloo W, African swine fever virus isolate, Georgia, 2007. Emerg Infect Dis. 2008;14:1870–4. 10.3201/eid1412.080591 19046509 PMC2634662

[5] Rowlands RJ, Duarte MM, Boinas F, Hutchings G, Dixon LK. The CD2v protein enhances African swine fever virus replication in the tick vector, *Ornithodoros erraticus.* Virology. 2009;393:319–28. 10.1016/j.virol.2009.07.040 19729182

[6] Basto AP, Nix RJ, Boinas F, Mencles S, Silva MJ, Cartaxeiro C, Kinetics of African swine fever virus infection in *Ornithodoros erraticus* ticks. J Gen Virol. 2006;87:1863–71. 10.1099/vir.0.81765-0 16760388

[7] Malmquist WA, Hay D. Hemadsorption and cytopathic effect produced by ASFV in swine bone marrow and buffy coat cultures. Am J Vet Res. 1960;21:104–8. 14420403

[8] Kleiboeker SB, Burrage TG, Scoles GA, Fish D, Rock DL. African swine fever virus infection in the argasid host, *Ornithodoros porcinus porcinus.* J Virol. 1998;72:1711–24. 9499019 10.1128/jvi.72.3.1711-1724.1998 PMC109458

[9] Kleiboeker SB, Scoles GA, Burrage TG, Sur JH. African swine fever virus replication in the midgut epithelium is required for infection of *Ornithodoros* ticks. J Virol. 1999;73:8587–98. 10482612 10.1128/jvi.73.10.8587-8598.1999 PMC112879

[10] Basto AP, Portugal RS, Nix RJ, Cartaxeiro C, Boinas F, Dixon LK, Development of a nested PCR and its internal control for the detection of African swine fever virus (ASFV) in *Ornithodoros erraticus.* Arch Virol. 2006;151:819–26. 10.1007/s00705-005-0654-2 16328146

